# Supplementary material for: Peptide-enhanced tough, resilient and adhesive eutectogels for highly reliable strain/pressure sensing under extreme conditions
Source: Nat Commun. 2022 Nov 5;13:6671. doi: 10.1038/s41467-022-34522-z (PMC9637226; doi:10.1038/s41467-022-34522-z)
Supplement: Supplementary file 1 — Supplementary information [file 41467_2022_34522_MOESM1_ESM.pdf]

Supplementary Information

Peptide-enhanced tough, resilient and adhesive eutectogels for highly reliable strain/pressure sensing under extreme conditions

Yan Zhang[1], Yafei Wang[1], Ying Guan[1,*] & Yongjun Zhang[2,*]

[1] Key Laboratory of Functional Polymer Materials, Institute of Polymer Chemistry, College of Chemistry, Nankai University, Tianjin 300071, P. R. China

[2] School of Chemistry, Tiangong University, Tianjin 300387, P. R. China

[*] Email: yingguan@nankai.edu.cn (Y. G.).

yongjunzhang@nankai.edu.cn (Y. Z.).

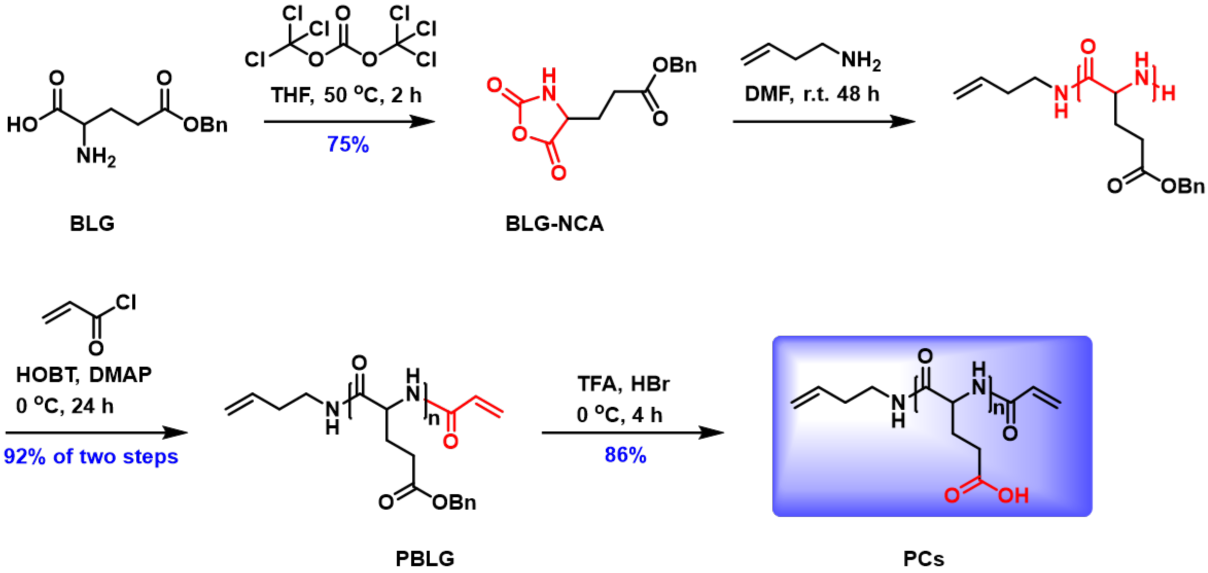

Supplementary Figure 1| Synthesis of the poly-L-glutamic acid-based PCs. Ring-opening polymerization of BLG-NCA initiated by 3-buten-1-amine, followed by capping the amino end with acryloyl chloride and deprotection of the benzyl groups gives the PCs.

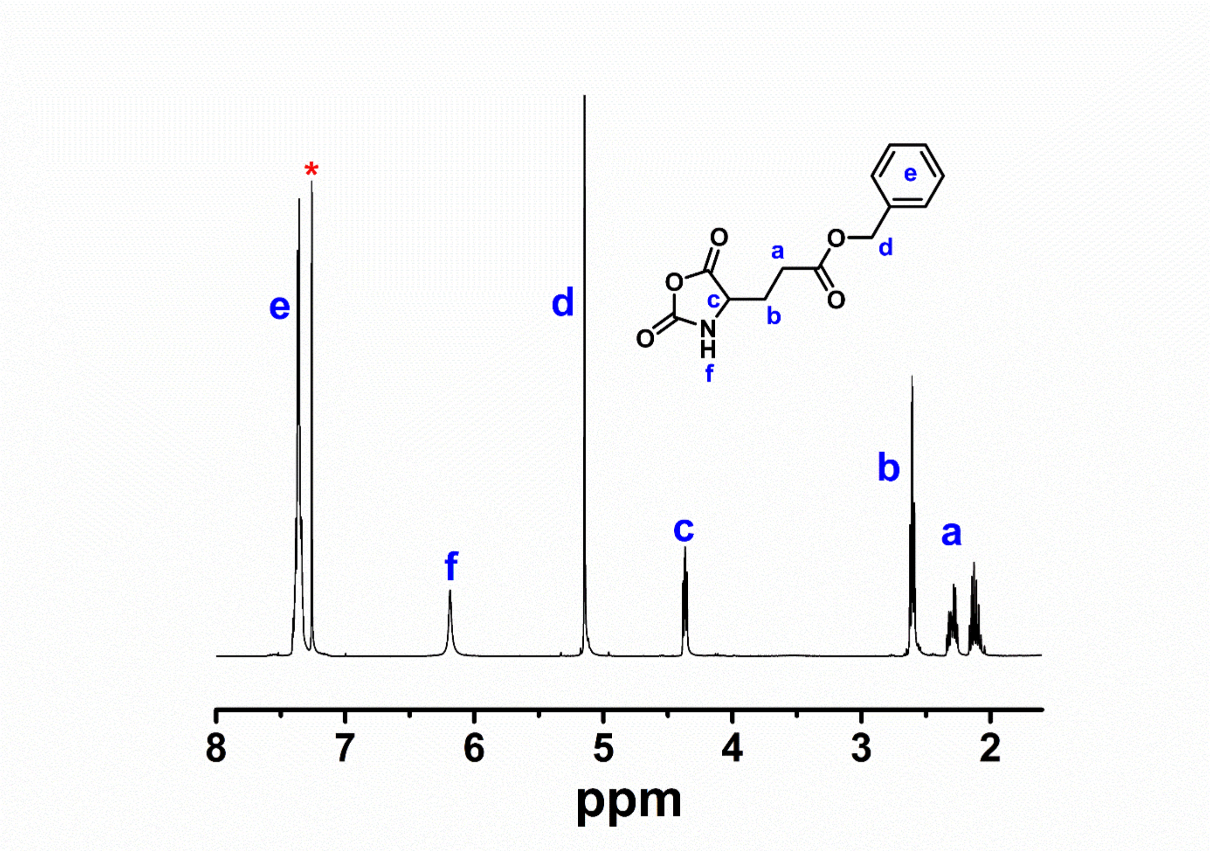

Supplementary Figure 2| ^{1}H NMR spectra of the BLG-NCA. The solvent is $CDCl_3$.

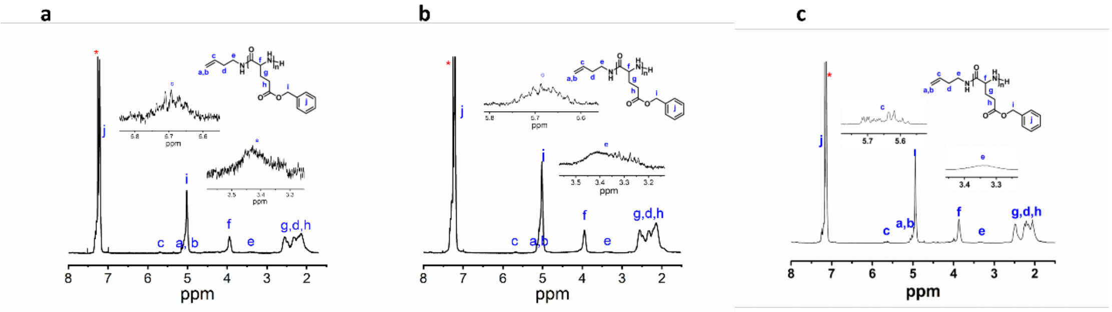

Supplementary Figure 3| ^{1}H NMR spectra of PBLG precursors of PC 12(a), PC 22 (b) and PC32 (c). The solvent is $CDCl_3$.

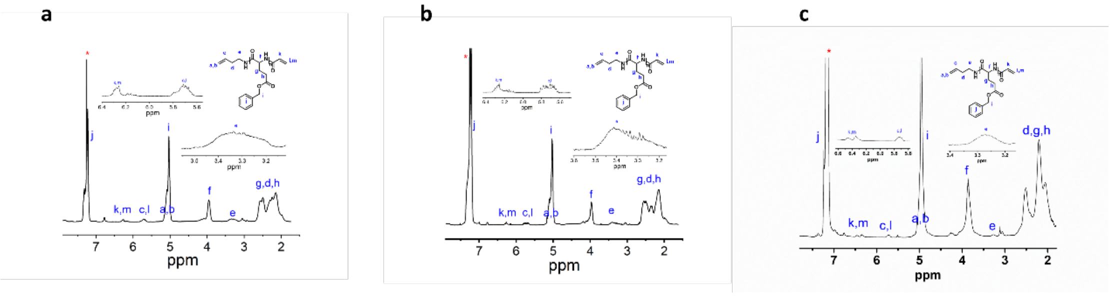

Supplementary Figure 4| ^{1}H NMR spectra of the acryl-terminated PBLG precursor of PC 12(a), PC 22 (b) and PC32 (c). The solvent is $CDCl_3$.

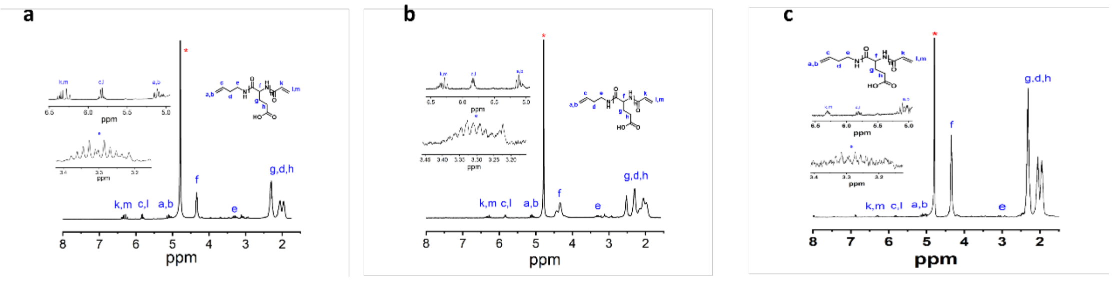

Supplementary Figure 5| ^{1}H NMR spectra of PC 12(a), PC 22 (b) and PC32 (c). The solvent is D_2O.

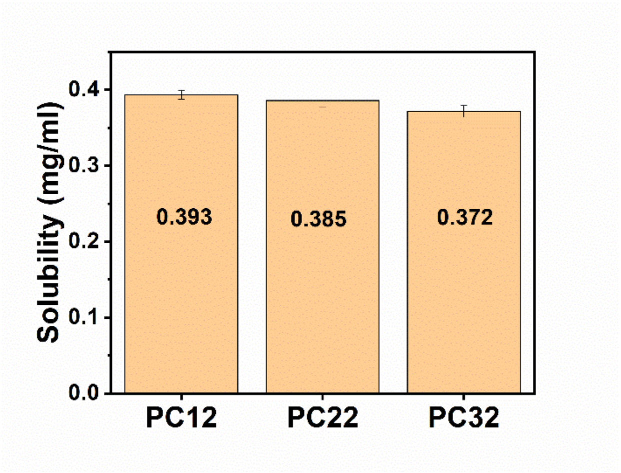

Supplementary Figure 6| Solubility of the PCs with different DPs in [ChCl][EG]. The error bars indicate standard deviation.

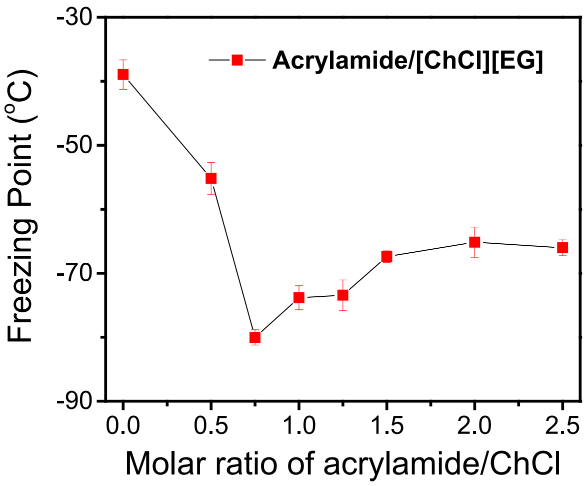

Supplementary Figure 7| Freezing point of acrylamide solutions in [ChCl][EG] as a function of molar ratio of acrylamide/ChCl. The error bars indicate standard deviation.

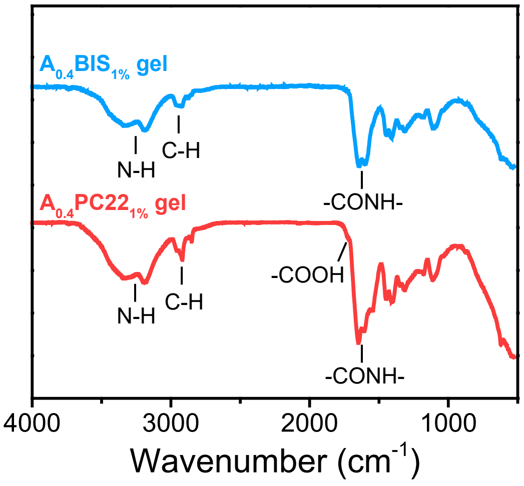

Supplementary Figure 8| FTIR spectra of $A_{0.4}PC22_{1\%}$ and $A_{0.4}BIS_{1\%}$ gels. The gels were synthesized in [ChCl][EG]. Before measurement, the gels were washed with DI water to remove the DES and dried.

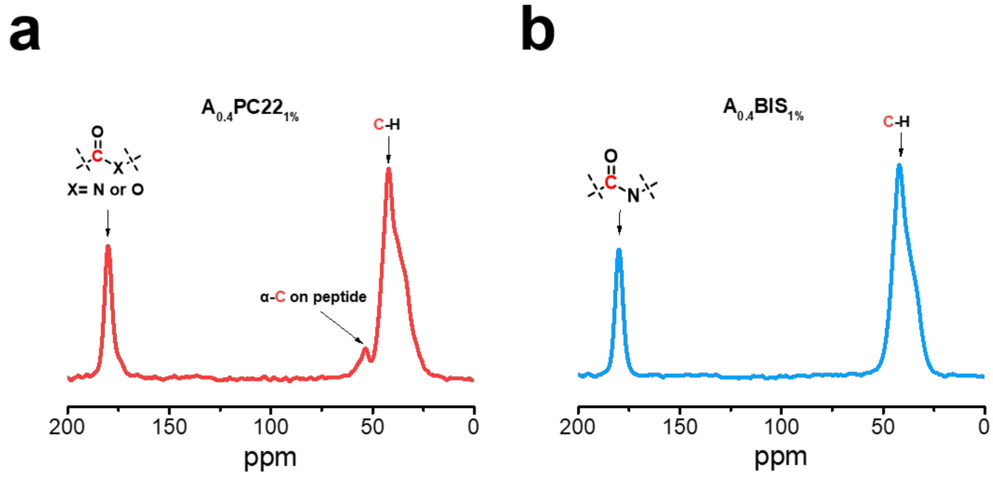

Supplementary Figure 9| ^{13}C solid-state NMR spectra of $A_{0.4}PC22_{1\%}$ (a) and $A_{0.4}BIS_{1\%}$ gels (b). The gels were synthesized in [ChCl][EG]. Before measurement, the gels were washed with DI water to remove the DES and dried.

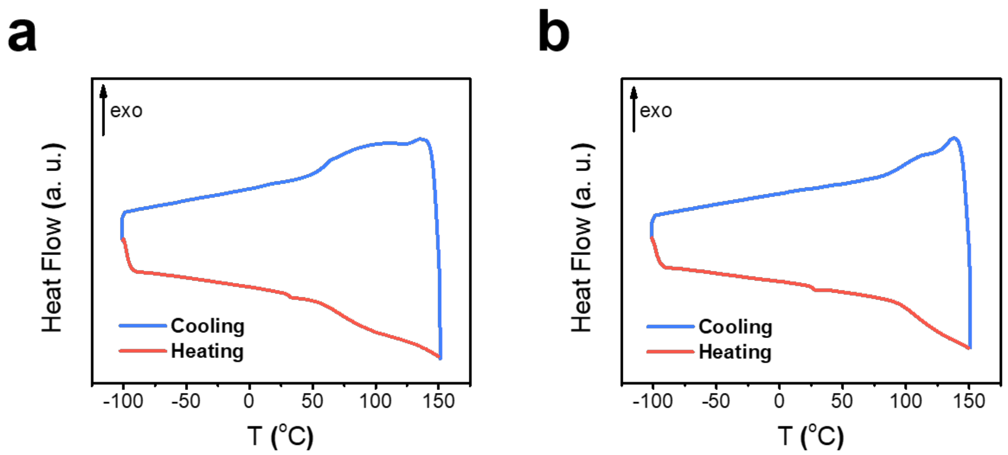

Supplementary Figure 10| DSC thermograms of $A_{0.4}PC22_{1\%}$ (a) and $A_{0.4}BIS_{1\%}$ gels (b). The gels were synthesized in [ChCl][EG]. Before measurement, the gels were washed with DI water to remove the DES and dried.

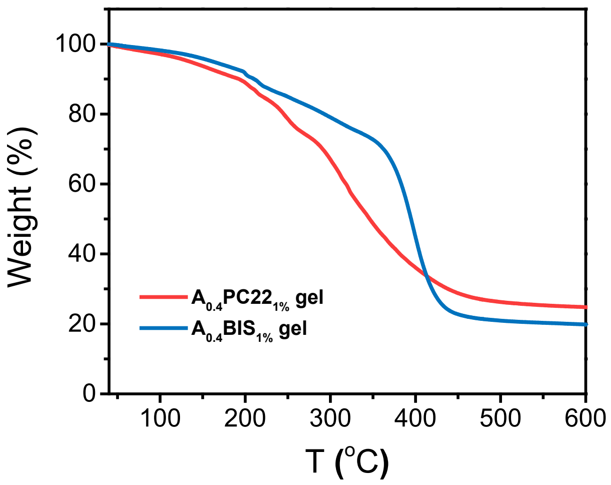

Supplementary Figure 11| TGA of $A_{0.4}PC22_{1\%}$ and $A_{0.4}BIS_{1\%}$ gels. The gels were synthesized in [ChCl][EG]. Before measurement, the gels were washed with DI water to remove the DES and dried.

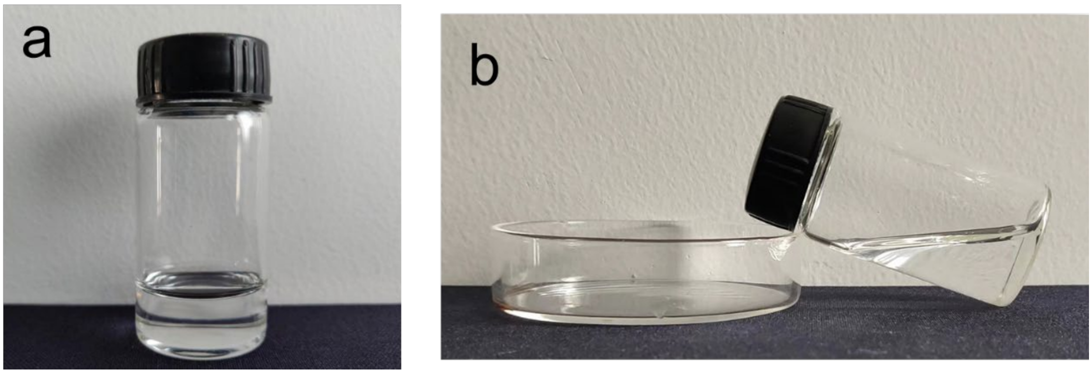

Supplementary Figure 12| Images of the pre-gel solution of $A_{0.4}PC22_{1\%}$ [ChCl][EG]. (a) Just prepared. (b) After 7 days storage in a refrigerator at 4 ºC.

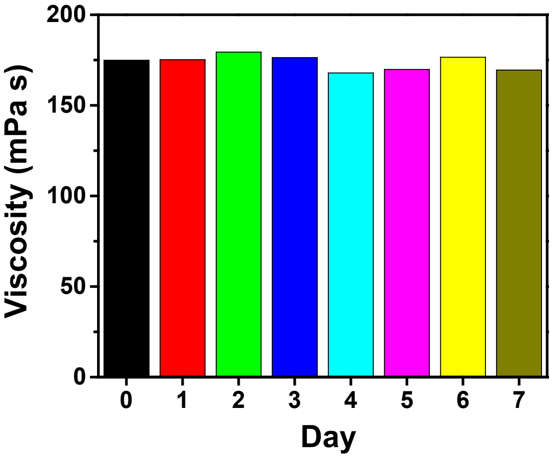

Supplementary Figure 13| Viscosity of the pre-gel solution of $A_{0.4}PC22_{1\%}$ [ChCl][EG]. The solution was stored in a refrigerator at 4 ºC.

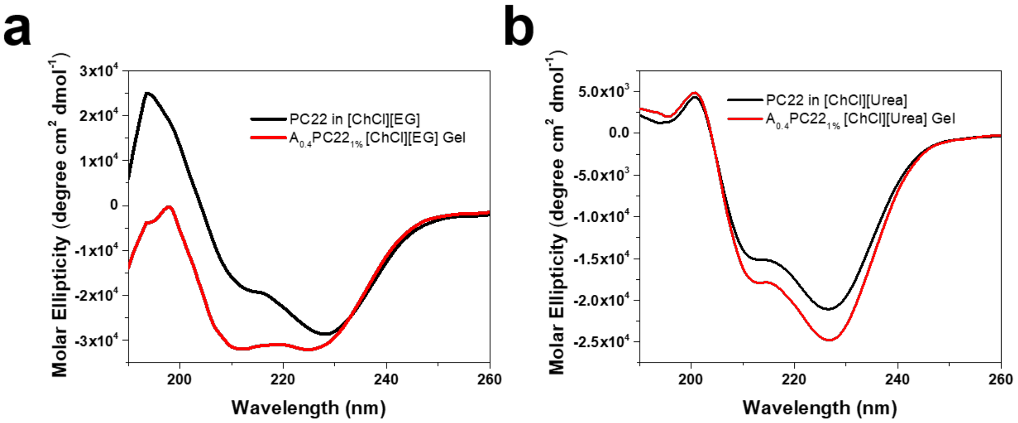

Supplementary Figure 14| CD spectra of PC22 solution in deep eutectic solvent and $A_{0.4}PC22_{1\%}$ gel in the same deep eutectic solvent. The deep eutectic solvent is [ChCl][EG] (a) and [ChCl][Urea] (b).

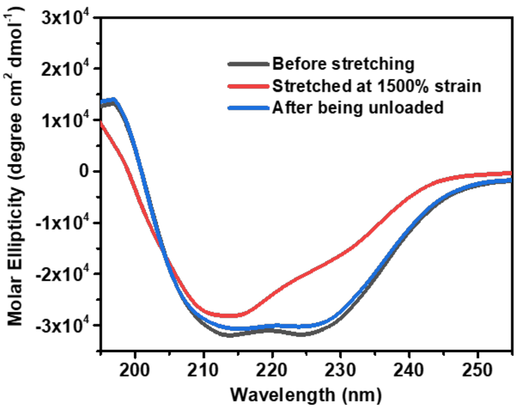

Supplementary Figure 15| CD spectra of an $A_{0.4}PC22_{1\%}$ [ChCl][EG] gel. The gel was stretched and then released.

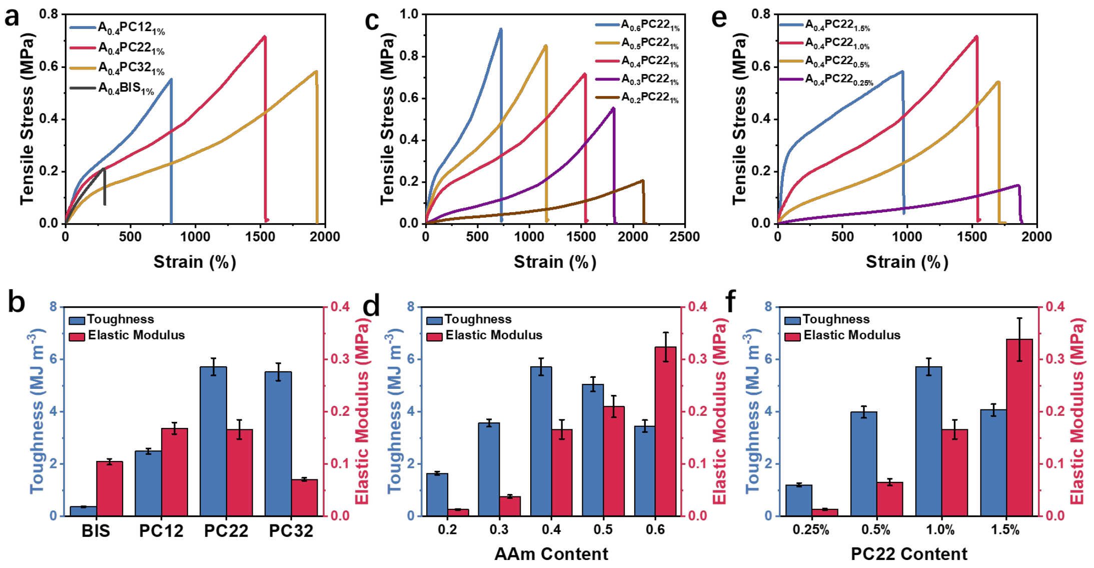

Supplementary Figure 16| Tensile properties of the $A_{0.4}PC22_{1\%}$ [ChCl][Urea] gels. **(a)** Tensile stress-strain curves and **(b)** mechanical properties of eutectogels crosslinked with different crosslinkers (PC12, PC22, PC32 and BIS). **(c)** Tensile stress-strain curves and **(d)** mechanical properties of the PC22-crosslinked eutectogels with different

concentrations of AAm (from 0.2 to 0.5) while the PC22 content is 1.0%. **(e)** Tensile stress-strain curves and **(f)** mechanical properties of eutectogels crosslinked with different concentrations of PC22 (from 0.25 to 1.5%) while the AAm content is 0.4. The error bars indicate standard deviation.

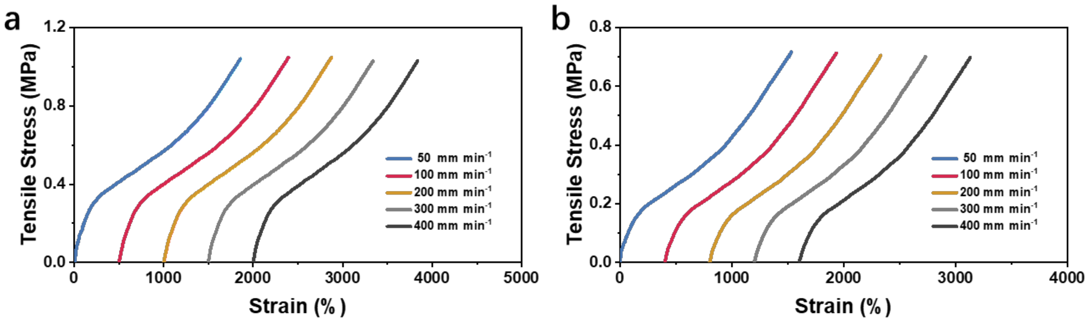

Supplementary Figure 17| Tensile tests at different tensile rates. Uniaxial tensile stress-strain curves of the $A_{0.4}PC22_{1\%}$ **(a)** [ChCl][EG] and **(b)** [ChCl][Urea] gels. The curves are shifted along x-axis for clarity.

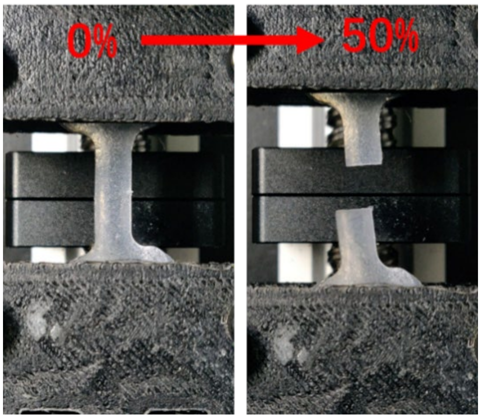

Supplementary Figure 18| Digital photos of a BIS cross-linked eutectogel in single edge crack test. The gel was stretched from 0% strain to 50% strain.

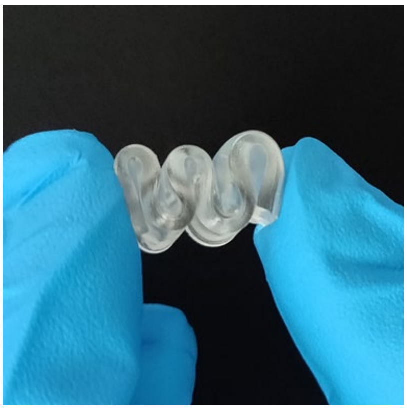

Supplementary Figure 19| Image of an $A_{0.4}PC22_{1\%}$ [ChCl][EG] gel. The gel retained significant flexibility after 3 days storage under medium vacuum conditions.

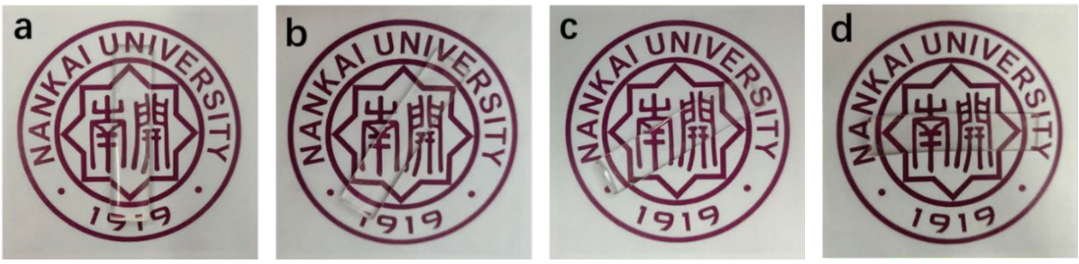

Supplementary Figure 20| Image of an $A_{0.4}PC22_{1\%}$ [ChCl][EG] gel. The gel was stored under medium vacuum condition (~40 Pa) for 0 h (a), 24 h (b), 48 h (c) and 72 h (d).

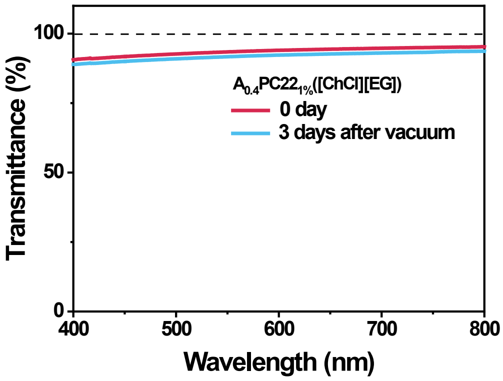

Supplementary Figure 21| Transmission spectra of an $A_{0.4}PC22_{1\%}$ [ChCl][EG] gel. The gel was stored under medium vacuum condition for 0 day and 3 days.

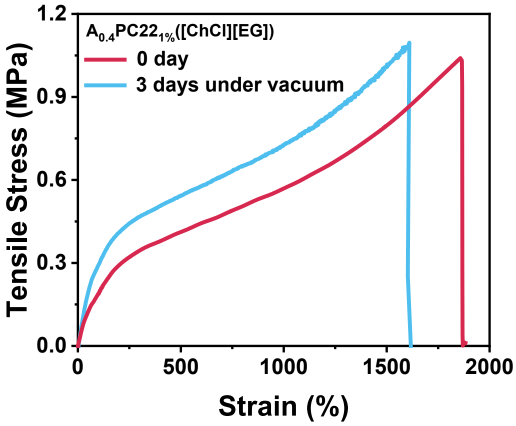

Supplementary Figure 22| Tensile stress-strain curves of an $A_{0.4}PC22_{1\%}$ [ChCl][EG] gel. The gel was stored under medium vacuum condition for 0 day and 3 days.

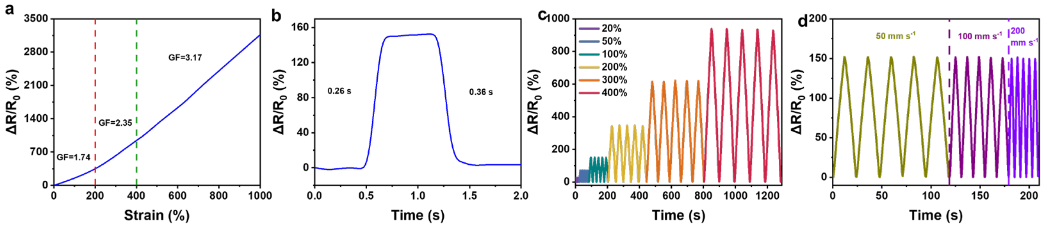

Supplementary Figure 23| Strain sensor application. (a) Relative resistance change of an $A_{0.4}PC22_{1\%}$ [ChCl][EG] gel as a function of applied strain. **(b)** Relative resistance change of the gel which was stretched to 200% strain, holded for a while and unloaded. **(c)** Repeated loading-unloading tests at 20%, 50%, 100%, 200%, 300% and 400% strain. The stretching rate was 100 mm s^{-1}. **(d)** Repeated loading-unloading tests at 100% strain with different stretching rates.

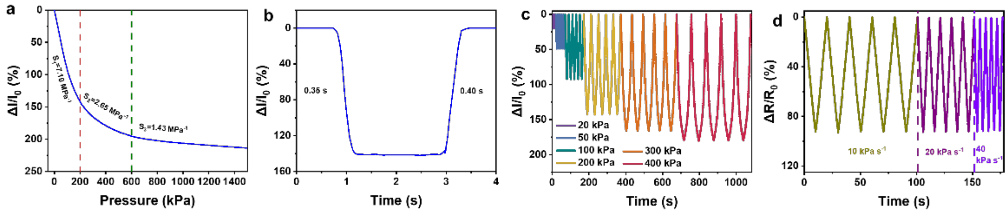

Supplementary Figure 24| Press sensor application. (a) Relative resistance change of an $A_{0.4}PC22_{1\%}$ [ChCl][EG] gel as a function of applied pressure. **(b)** Relative

resistance change of the gel which was pressed to 200 kPa, held for a while and then unloaded. **(c)** Repeated loading-unloading tests at 20, 50, 100, 200, 300 and 400 kPa pressure. The pressing rate was 20 kPa s^{-1}. **(d)** Repeated loading-unloading tests at 100 kPa pressure with different pressing rates.

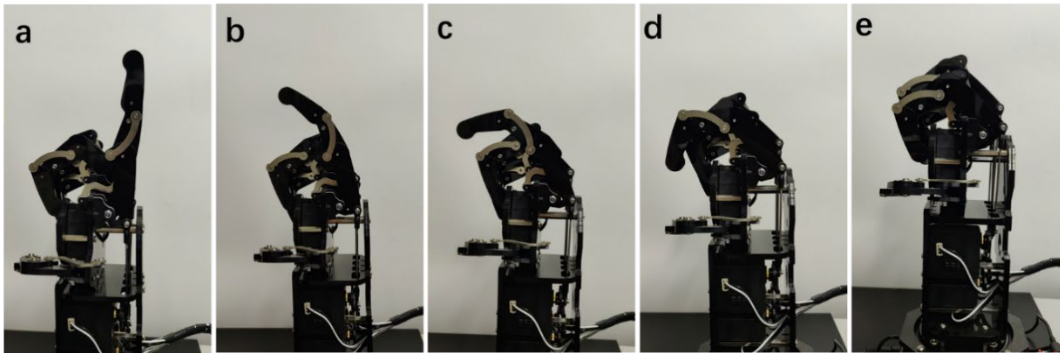

Supplementary Figure 25| Position of robotic fingers at diverse PWM signal value of the servo motor. (a) 2500. (b) 2000. (c) 1500. (d) 1000. (e) 500.

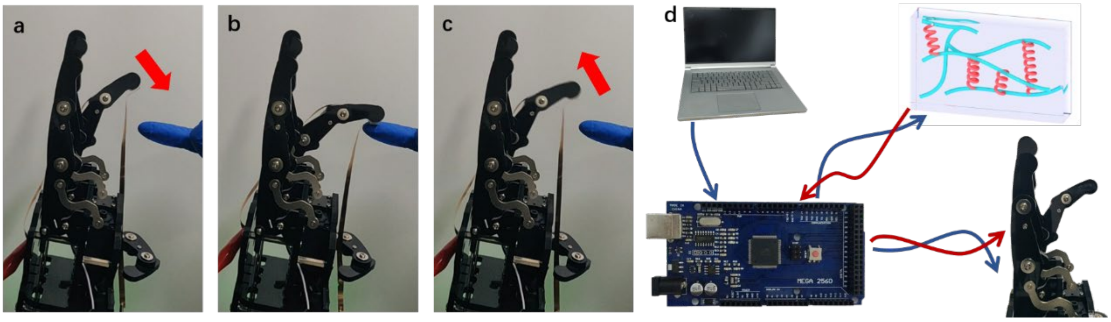

Supplementary Figure 26| Robotic finger intelligent obstacle avoidance. The mechanical fingers that were **(a)** bending, **(b)** touching the human finger, and finally **(c)** rising quickly to avoid the collision. **(d)** Principle of function implementation. The blue arrow represented the control flow during normal operation, and the red arrow represented the control flow when it touched an obstacle.

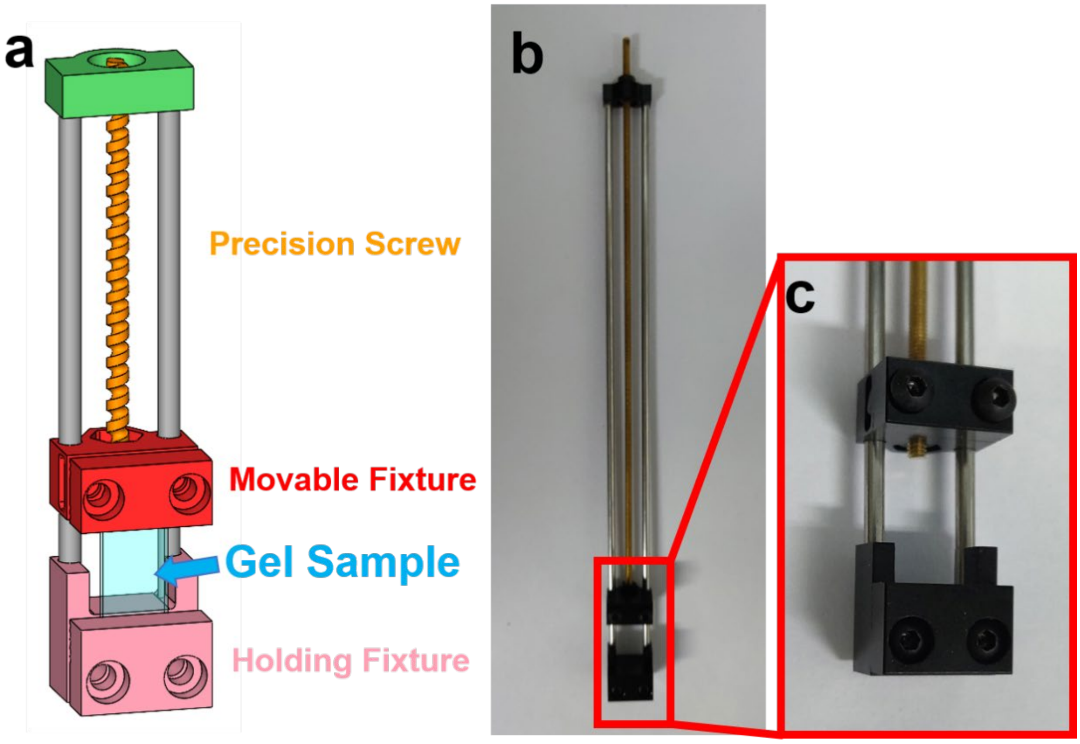

Supplementary Figure 27| The home-made stretching device used to measure CD spectra of stretched gels. (a) The schematic diagram. (b, c) The photograph of the device.

Supplementary Table 1| Synthesis and characterization of peptide crosslinkers.

Sample	Monomer/Initiator	Mn [a]	Mw [a]	Mw/Mn [a]	DP(GPC) [a]	DP(NMR) [b]
PC12	15	2835	2927	1.032	12.4	11.6
PC22	25	4986	5212	1.045	22.2	21.2
PC32	35	7154	7605	1.063	32.1	31.0

[a] Measured by GPC. Mn and Mw are number average molecular weight and weight average molecular weight of the PBGA precusors. [b] Measured by ^{1}H NMR.

Supplementary Table 2| Dissolution of PC22 in DESs.

HBA	HBD	HBA:HBD molar ratio	Viscosity (cP) at 298 K	Dissolution (RT)	Dissolution (80 °C)
ChCl	EG	1:2	48 [1]	Good	Better
ChCl	Glycerol	1:2	281 [2]	Good	Better
ChCl	Urea	1:2	750 [3]	Good	Better
ChCl	Ethanedioic Acid	1:1	597 [4]	Poor	Improve a little
ChCl	Glutaric Acid	1:1	2015 [5]	Poor	Great improvement
ChCl	Glucose	1:1	34400 [6]	Poor	Poor

HBA and HBD represent hydrogen bond acceptor and hydrogen bond donor, respectively.

Supplementary Table 3| Comparison of fracture toughness between $A_{0.4}PC22_{1\%}$ and previously reported eutectogels.

Sample	Polymer content	Resilience (Strain)	Toughness (kJ m^{-3})	Fracture energy (J m^{-2})	Reference
SR-0.38	38%	98% (1100%)	22000	3600	[7]
Tetra-0.33	33%	100% (300%)	550	3600	[7]
DFT-10-7	12%	49% (300%)	1300	280	[8]
Crystallized PVA-PAAm	30%	45% (250%)	1800	2900	[9]
PAM-CS-A	21%	36% (400%)	10000	13000	[10]
s-gel (AA/AM = 0.2)	49%	34% (200%)	17000	4800	[11]
Hybrid	14%	29% (700%)	2400	8700	[12]
MM-0.2-6	53%	21% (200%)	31000	19000	[13]
$A_{0.4}PC22_{1\%}$	46%	91.6% (500%)	10600	13600	This work

Resilience was represented by energy dissipation, which was defined as the ratio of the area covered by the second cycle loading curve to that covered by the first one.

Supplementary Table 4| Comparison of mechanical properties of $A_{0.4}PC22_{1\%}$ [ChCl][EG] gel with previously reported eutectogels.

HBA	HBD	HBA:HBD molar ratio	Gel network components	Elongation at break	Strength at break	Resilience	Reference
ChCl	EG	1:2	Acrylic acid/$AlCl_3$/Cellulose	1100%	1.23 MPa	42.9% (300% strain; Waiting 15 min)*	[14]
ChCl	Urea/glycerol	1:2/0.125	AAm/Cellulose/BIS	~280%	55.5 kPa		[15]
ChCl	EG	1:2	Gelatin	320%	67 kPa	~92.5% (165% strain)**	[16]
ChCl	Acrylic acid	1:2	Acrylic acid/PEGDA200	300%	500 kPa		[17]
betaine	EG	1:3	Acrylic acid/FeCl3	2600%	50 kPa		[18]
ChCl	EG	1:2	Acrylic acid/Sulfobetaine vinylimidazole/ PEGDA575	680%	64 kPa	95% (300% strain; Waiting 5 min)**	[19]
ChCl	Propane-1,3-Diol	1:2	(R)-12-hydroxystearic acid hydrazide/ *N*-hydroxyethylacrylamide/BIS	4400%	0.26 MPa	74.7%*	[20]
ChCl	EG	1:2	Bisgluconamide derivatives/*N*-Hydroxyethyl acrylamide/BIS	5600%	0.37 MPa	62.2%*	[21]
ChCl	Acrylic acid/AAm	1:1/1	Acrylic acid/AAm/BIS	~310%	125.87 kPa		[22]
ChCl	Acrylic acid	1:2	Acrylic acid/eGaIn	~1050%	~255 kPa	Hysteresis can be observed	[23]
ChCl	Glycerol	1:2	Polyurethane/Tannic acid	~220%	~1.13 MPa	Hysteresis can be observed	[24]
ChCl	EG	1:2	DMAPS/2-Hydroxyethyl/ Methacrylate/PEGDA550	527%	204.5 kPa	Hysteresis can be observed	[25]
ChCl	EG	1:2	PVA-1799/Acrylic acid/Phytic acid	680%	2.6 MPa	96% (Waiting 10 s)***	[26]
ChCl	Urea	1:2	AAm	~390%	2.2 MPa		[27]
[BMIM]Cl	Acrylic acid	1:3	Bacterial cellulose	94%	~0.4 MPa		[28]
ChCl	Acrylic acid	1:3	Bacterial cellulose	175%	~0.55 MPa		[28]
LiTFSI	*N*-methylacetamide	1:4	PVDF-HFP	300%	~0.6 MPa		[29]
ChCl	Glycerol	1:2	Acrylic acid/PEGDA	~1530%	~200 kPa		[30]
ChCl	Urea	1:2	Acrylic acid/PEGDA	~1130%	~310 kPa		[30]
ChCl	Diethylene glycol	1:2	Acrylic acid/PEGDA	~2300%	~180 kPa		[30]
ChCl	EG	1:2	Acrylic acid/PEGDA	~1200%	~150 kPa	~80% (100% strain; Waiting 1 min)***	[30]
ChCl	EG	1:2	AAm/PGlu	1860%	1.04 MPa	~91.6% (First cycle; 500% strain; Immediately)* ~91.0% (First cycle; 500% strain; Immediately)**	This work
ChCl	Urea	1:2	AAm/PGlu	1540%	0.72 MPa	~90.4% (First cycle; 500% strain; Immediately)* ~89.3% (First cycle; 500% strain; Immediately)**	This work

*, ** and *** denote the resilience represented by energy dissipation, strain or stress, respectively.

Supplementary References:

1. Y. Zhang, et al. Liquid Structure and Transport Properties of the Deep Eutectic Solvent Ethaline. *J. Phys. Chem. B* **124**, 5251-5264 (2020).
2. M. K. AlOmar, et al. Glycerol-based deep eutectic solvents: Physical properties. *J. Mol. Liq.* **215**, 98-103 (2016).
3. F. S. Mjalli & N. M. Abdel Jabbar. Acoustic investigation of choline chloride based ionic liquids analogs. *Fluid Phase Equilib.* **381**, 71-76 (2014).
4. D. B. Andrew P. Abbott, Glen Capper, David L. Davies, Raymond K. Rasheed. Deep eutectic solvents formed between choline chloride and carboxylic acids: versatile alternatives to ionic liquids. *J. Am. Chem. Soc.* **126**, 9142-9147 (2004).
5. C. Florindo, et al. Insights into the synthesis and properties of deep eutectic solvents based on cholinium chloride and carboxylic acids. *ACS Sustainable Chem. Eng.* **2**, 2416-2425 (2014).
6. Z. Maugeri & P. Domínguez de María. Novel choline chloride based deep eutectic solvents with renewable hydrogen bond donors: levulinic acid and sugar-based polyols. *RSC Adv.* **2**, 421-425 (2012).
7. C. Liu, et al. Tough hydrogels with rapid self-reinforcement. *Science* **372**, 1078-1081 (2021).
8. L. Zhang, et al. Anisotropic tough poly(vinyl alcohol) hydrogels. *Soft Matter* **8**, 10439-10447 (2012).
9. J. Li, Z. Suo&J. J. Vlassak. Stiff, strong, and tough hydrogels with good chemical stability. *J. Mater. Chem. B* **2**, 6708-6713 (2014).
10. Y. Yang, et al. A universal soaking strategy to convert composite hydrogels into extremely tough and rapidly recoverable double-network hydrogels. *Adv. Mater.* **28**, 7178-7184 (2016).
11. Y. Liang, J. Xue, B. Du & J. Nie. Ultrastiff, tough, and healable ionic–hydrogen bond cross-linked hydrogels and their uses as building blocks to construct complex hydrogel structures. *ACS Appl. Mater. Interfaces* **11**, 5441-5454 (2019).
12. J. Y. Sun, et al. Highly stretchable and tough hydrogels. *Nature* **489**, 133-6 (2012).
13. Y. J. Wang, et al. Ultrastiff and tough supramolecular hydrogels with a dense and robust hydrogen bond network. *Chem. Mater.* **31**, 1430-1440 (2019).
14. C.-W. Lai & S.-S. Yu. 3D printable strain sensors from deep eutectic solvents and cellulose nanocrystals. *ACS Appl. Mater. Interfaces* **12**, 34235-34244 (2020).
15. S. Hong, et al. A stretchable and compressible ion gel based on a deep eutectic solvent applied as a strain sensor and electrolyte for supercapacitors. *J. Mater. Chem. C* **8**, 550-560 (2020).
16. H. Qin, R. E. Owyeung, S. R. Sonkusale & M. J. Panzer. Highly stretchable and nonvolatile gelatin-supported deep eutectic solvent gel electrolyte-based ionic skins for strain and pressure sensing. *J. Mater. Chem. C* **7**, 601-608 (2019).
17. X. Wang, et al. Weavable transparent conductive fibers with harsh environment tolerance. *ACS Appl. Mater. Interfaces* **13**, 8952-8959 (2021).
18. J. Wang, et al. Ultra-stretchable, self-healing, conductive, and transparent PAA/DES ionic gel. *Macromol. Rapid Commun.* **42**, 2000445 (2021).
19. X. Bu, et al. Design of highly stretchable deep eutectic solvent-based ionic gel electrolyte with high ionic conductivity by the addition of zwitterion ion dissociators for flexible supercapacitor. *Polym. Eng. Sci.* **61**, 154-166 (2021).
20. Y. Liang, et al. Low-molecular-weight supramolecular-polymer double-network eutectogels for self-adhesive and bidirectional sensors. *Adv. Funct. Mater.* **31**,

2104963 (2021).
21. K. Wang, et al. Super-stretchable and extreme temperature-tolerant supramolecular-polymer double-network eutectogels with ultrafast in situ adhesion and flexible electrochromic behaviour. *Mater. Horiz.* **8**, 2520-2532 (2021).
22. Y. Chen, S. Li&S. Yan Starch as a reinforcement agent for poly(ionic liquid) hydrogels from deep eutectic solvent via frontal polymerization. *Carbohydr. Polym.* **263**, 117996 (2021).
23. M. Wang, et al. Multifunctional liquid-free ionic conductive elastomer fabricated by liquid metal induced polymerization. *Adv. Funct. Mater.* **31**, 2101957 (2021).
24. S. Wang, et al. Self-adhesive, stretchable, biocompatible, and conductive nonvolatile eutectogels as wearable conformal strain and pressure sensors and biopotential electrodes for precise health monitoring. *ACS Appl. Mater. Interfaces* **13**, 20735-20745 (2021).
25. J. Lan, et al. Zwitterionic dual-network strategy for highly stretchable and transparent ionic conductor. *Polymer* **231**, 124111 (2021).
26. Y. Wang, J. Wang, Z. Ma & L. Yan. A highly conductive, self-recoverable, and strong eutectogel of a deep eutectic solvent with polymer crystalline domain regulation. *ACS Appl. Mater. Interfaces* **13**, 54409-54416 (2021).
27. Q. Zhou, et al. Versatile ionic gel driven by dual hydrogen bond networks: toward advanced lubrication and self-healing. *ACS Appl. Polym. Mater.* **3**, 5932-5941 (2021).
28. M. A. Smirnov, et al. Polymerizable choline- and imidazolium-based ionic liquids reinforced with bacterial cellulose for 3D-printing. *Polymers* **13**, 3044 (2021).
29. Z. Li, et al. Deep eutectic solvent-immobilized PVDF-HFP eutectogel as solid electrolyte for safe lithium metal battery. *Mater. Chem. Phys.* **267**, 124701 (2021).
30. G. Li, et al. A stretchable and adhesive ionic conductor based on polyacrylic acid and deep eutectic solvents. *npj Flex. Electron.* **5**, 23 (2021).
