# Supplementary material for: Peptide-enhanced tough, resilient and adhesive eutectogels for highly reliable strain/pressure sensing under extreme conditions
Source: Nat Commun. 2022 Nov 5;13:6671. doi: 10.1038/s41467-022-34522-z (PMC9637226; doi:10.1038/s41467-022-34522-z)
Supplement: Supplementary file 3 — Description of Additional Supplementary Files [file 41467_2022_34522_MOESM3_ESM.pdf]

Description of Additional Supplementary Files

File Name: Supplementary Movie 1
Description: Pressing a $A_{0.4}PC22_{1\%}$ [ChCl][EG] gel film firmly against a sharp tip (0.5 mm in diameter).

File Name: Supplementary Movie 2
Description: Stretching an $A_{0.4}PC22_{1\%}$ [ChCl][EG] gel sample with a 2.5 mm crack. The width of the gel was 5.0 mm. The crack was stained with rhodamine B for visibility.

File Name: Supplementary Movie 3
Description: Stretching an $A_{0.4}BIS_{1\%}$ [ChCl][EG] gel with a 0.5 mm crack.

File Name: Supplementary Movie 4
Description: Twisting an $A_{0.4}PC22_{1\%}$ [ChCl][EG] gel after cooled to -27 °C.

File Name: Supplementary Movie 5
Description: A moving robotic finger installed with gel sensor touched a human finger and quickly lift up to avoid it, while a moving robotic finger without gel sensor continued to move when touched a human finger.
